# Low Specificity of Determine HIV1/2 RDT Using Whole Blood in South West Tanzania

**DOI:** 10.1371/journal.pone.0039529

**Published:** 2012-06-29

**Authors:** Inge Kroidl, Petra Clowes, Wolfram Mwalongo, Lucas Maganga, Leonard Maboko, Arne L. Kroidl, Christof Geldmacher, Harun Machibya, Michael Hoelscher, Elmar Saathoff

**Affiliations:** 1 Division of Infectious Diseases and Tropical Medicine, Medical Center of the University of Munich (LMU), Munich, Germany; 2 NIMR-Mbeya Medical Research Programme, Mbeya, Tanzania; 3 German Centre for Infection Research, LMU, Munich, Germany; 4 Mbeya Regional Medical Office, Mbeya, Tanzania; Tulane University, United States of America

## Abstract

**Objective:**

To evaluate the diagnostic performance of two rapid detection tests (RDTs) for HIV 1/2 in plasma and in whole blood samples.

**Methods:**

More than 15,000 study subjects above the age of two years participated in two rounds of a cohort study to determine the prevalence of HIV. HIV testing was performed using the Determine HIV 1/2 test (Abbott) in the first (2006/2007) and the HIV 1/2 STAT-PAK Dipstick Assay (Chembio) in the second round (2007/2008) of the survey. Positive results were classified into faint and strong bands depending on the visual appearance of the test strip and confirmed by ELISA and Western blot.

**Results:**

The sensitivity and specificity of the Determine RDT were 100% (95% confidence interval  = 86.8 to 100%) and 96.8% (95.9 to 97.6%) in whole blood and 100% (99.7 to 100%) and 97.9% (97.6 to 98.1%) in plasma respectively. Specificity was highly dependent on the tested sample type: when using whole blood, 67.1% of positive results were false positive, as opposed to 17.4% in plasma. Test strips with only faint positive bands were more often false positive than strips showing strong bands and were more common in whole blood than in plasma. Evaluation of the STAT-PAK RDT in plasma during the second year resulted in a sensitivity of 99.7% (99.1 to 99.9%) and a specificity of 99.3% (99.1 to 99.4%) with 6.9% of the positive results being false.

**Conclusions:**

Our study shows that the Determine HIV 1/2 strip test with its high sensitivity is an excellent tool to screen for HIV infection, but that – at least in our setting – it can not be recommended as a confirmatory test in VCT campaigns where whole blood is used.

## Introduction

The World Health Organisation (WHO) Global Programme on AIDS first recommended the use of simple rapid detection tests (RDTs) for HIV in 1992 [1]. Since then RDTs have been widely used throughout Africa and the developing world for blood safety screening, surveillance and in prenatal or voluntary counselling and testing (VCT) centres [2]. Until recently the National guidelines for VCT in Tanzania recommended using the SD Bioline HIV 1/2 3.0 (Standard Diagnostics, Kyonggi-do, Korea) as a screening test, with initial reactive samples re-tested using the Determine HIV 1/2 test (Abbott Laboratories, Abbott Park, IL). The Uni-Gold™ HIV-1/2 (Trinity Biotech, Bray Ireland) is used as a final test if results are discrepant [3]. A fourth HIV rapid test, HIV 1/2 STAT-PAK (Chembio Diagnostics Systems, Medford, NY, USA) is used in neighbouring countries mainly as a confirmatory test. All four RDTs are less expensive than ELISA or Western Blot tests, require little or no equipment, can be stored at room temperature and are easy to use and to read. Because they allow for real-time, point-of-care HIV testing, individuals can receive their test result during a single clinic visit which is likely to increase the uptake of VCT [4], [5], especially in Sub Saharan Africa, where in some settings more than two weeks may be needed for laboratory results to become available [6]. However, it seems that the diagnosis of HIV by RDT is challenged by confounding regional factors. Especially the Determine HIV 1/2 test received very diverse reports regarding its specificity which ranged from 91.7% to above 99% [7], [8], [9], [10], [11], [12].

Here we evaluate the diagnostic test performance for HIV testing of the Determine HIV 1/2 and the HIV 1/2 STAT-PAK (Chembio Diagnostics Systems, Medford, NY, USA) RDTs in a general population cohort in South West Tanzania where, in contrast to previous studies, children and adults of both genders were included. The two tests are referred to below as the “Determine” RDT and the “STAT-PAK” RDT, in order to improve the readability of this article. The manufacturer’s information applies to the time of the study; today both tests are manufactured and sold by Alere, formerly named Inverness Medical Professional Diagnostics, Princeton, NJ.

## Methods

### Ethical Considerations

The study was approved by the National Ethical Committee/Medical Research Coordinating Committee of the National Institute for Medical Research, Tanzania. All adult participants provided written informed consent prior to enrolment into the study. Parents gave the permission for their children, if they were below 18 years of age. In addition children above the age of 12 years signed their own consent form together with their parents. The study, including the consent forms for adults and children, was additionally approved by the Mbeya Medical Research and Ethics Committee.

### Data Collection

Data for this study were collected between May 2006 and June 2008 in the Mbeya Region in Tanzania. The EMINI project (Establishment of the infrastructure to **E**valuate and **M**onitor the **I**mpact of **N**ew **I**nterventions), a population based cohort study, investigated the prevalence and incidence of HIV in 9 selected communities with a total population of ∼171,000 people. The urban, semi-urban and rural study sites were carefully chosen in order to reflect the geographic diversity of the Region, from altitudes of about 475 meters above sea level near Lake Nyassa, to over 2300 meters. Subsequently, 10% of all households in the study sites were randomly selected, which resulted in a cohort of approximately 18,000 individuals of all age groups and both sexes. Study participants were visited annually for specimen and data collection, including an HIV rapid test. Collected data further included socio-economic characteristics of the study participants, previous and current medical history, as well as individual knowledge regarding different diseases.

During the first survey (2006/2007) the Determine RDT was used to screen for HIV-1 infection. 2.7 ml of blood were collected by venae puncture from participants above the age of 6 years. However, venae puncture was not possible in all participants, and especially problematic in younger children. In these cases, 300 µl of blood were collected after finger prick, using microvettes (Sarstedt, Nürnbrecht, Germany). In most cases this smaller amount of blood did not supply sufficient plasma, so that HIV testing in children below 6 years was mostly done using whole blood. During the second and third annual survey round, where the STAT-PAK RDT was used for HIV screening, we collected 2.7 ml of blood from all participants above 2 years of age, thus all our STAT-PAK RDT results were obtained from plasma samples. Results of the third survey are not reported here, they were only used when needed to verify results from the two previous surveys. For both RDTs our results only include data from participants above the age of 2 years in order to exclude false test-positivity due to the presence of maternal antibodies.

According to the manufacturer’s instructions for both assays, any test result with visible lines in both test control areas, regardless of intensity, were considered reactive [13], [14]. However, we recorded variations of intensity of the strip test. Any assay with an apparent positive band which was noticeably lighter than the control band on the test card was regarded as “faint”.

Additional testing to verify the initial RDT results was only done if.

- the RDT result was the first HIV positive result obtained from this participant (true for positive results from survey 1 and newly positive results from survey 2), or if
- the RDT result differed from the result of the previous or the following survey.

**Figure 1:**
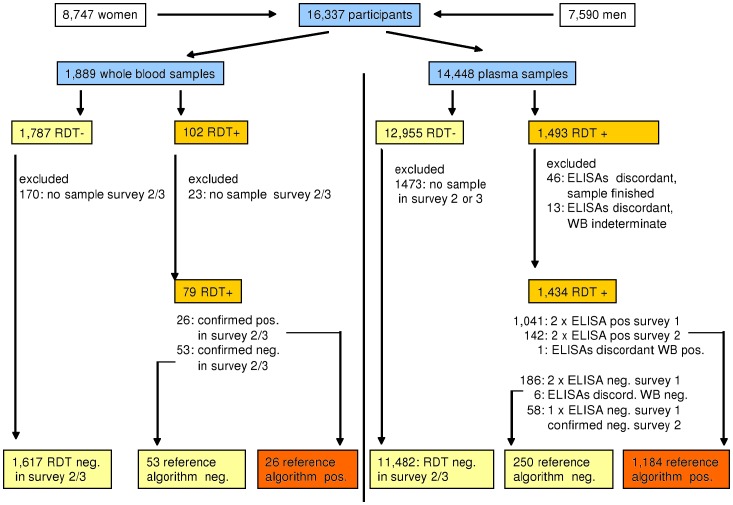
HIV-Testing Algorithm and Exclusion criteria Survey 1. In Survey 1 we tested 1,889 whole blood specimen and 14,448 plasma specimen with the Determine HIV1/2 RDT. The confirmation algorithm included two different ELISAs and one Western Blot. If both ELISAs were in agreement their result was used as the reference standard result. Samples with discordant ELISA results were retested by Western Blot and the Western Blot result used. Samples with indeterminate Western Blot results were excluded from analysis. Negative RDT results were not directly confirmed, but regarded as true negative if the result of the following survey was also negative. Whole blood results where confirmation by ELISA testing was impossible due to lack of plasma, were regarded as true positive if the result in the next survey was confirmed positive and as false positive if a negative test result in the next survey was confirmed using the above reference algorithm. Results where the true HIV status could not be verified according to the reference algorithm were excluded from this analysis.

**Figure 2:**
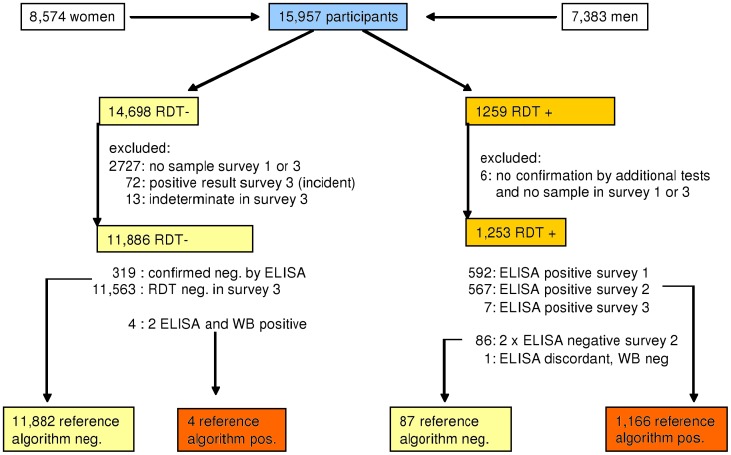
HIV-Testing Algorithm and Exclusion criteria Survey 2. In Survey 2 we tested only plasma samples from 15,957 participants using the STAT-PAK-HIV1/2 RDT. The confirmation algorithm was essentially the same as in Survey 1.It included two different ELISAs and one Western Blot for RDT positive samples. Participants, who’s positive RDT result had been confirmed in Survey 1 already were not re-tested. 319 negative RDT results were directly confirmed by Behring ELISA, 11563 were regarded as true negative because the result of the following survey was also negative.

In these cases the sample was retested using an ELISA HIV test (Enzygnost Anti HIV 1/2 Plus, DADE-Behring, Marburg, Germany) (Figures 1 and 2). Samples where the RDT and the ELISA had discordant results were tested again, using a second ELISA (rLAV, Biorad Laboratories, Redmond WA, USA). If both ELISAs were in agreement, the ELISA result was used as the reference-standard. Samples with discordant ELISA results were retested by Western Blot (MPD HIV Blot 2.2, MP Biomedicals, and Geneva, Switzerland) to determine the HIV status. If the Western Blot result was indeterminate, the results for this sample were excluded from statistical analysis. In addition, samples with discordant results were repeat-tested with the respective RDT to reconfirm the initial RDT results and to exclude a technical error.

Retesting of whole blood results was not possible due to lack of stored plasma. Instead we regarded these results as true positive if the result in the next survey was also positive and as false positive if the participant had a negative test result in the subsequent survey, which was confirmed using the above reference algorithm. Whole blood results from participants not participating in at least one of the two following surveys were excluded from our analysis because their true HIV status could not be determined.

Negative RDTs from the first or second annual surveys which were confirmed by another negative RDT in the following survey and were not in conflict with earlier positive results, were regarded as confirmed and not further tested. For all HIV incident cases, which were found newly positive in surveys 2 or 3, the negative result of the previous round was confirmed by the above described testing reference algorithm.

All participants from the nine different study sites were visited at their home. The collected specimens were brought to the central laboratory in Mbeya, where the RDTs were performed in order to ascertain comparable test quality across sites and field teams. In addition to HIV other concomitant infections and influences were examined. *Plasmodium falciparum* infection was assessed using Rapid Malaria Tests (ICT Diagnostics, Cape Town, South Africa) and *Schistosoma haematobium* infection was assessed by urine microscopy for *S. haematobium* eggs.

Information on elevation was retrieved using data from the NASA Shuttle Radar Topography Mission (SRTM) global digital elevation model (DEM) version 2.1 with a nominal resolution of 90 meters. Annual ambient temperature interpolated surfaces with 1 km spatial resolution were downloaded from the WorldClim – Global Climate Data website (http://www.worldclim.org/) [15], [16]. These data and the participant household’s positions which had been collected using handheld GPS receivers were combined in a geographic information system in order to calculate elevation and mean annual ambient temperature at the household position.

### Statistical Analysis

All statistical analyses were performed using Stata version 10 (Stata Corp., College Station, TX). Diagnostic test performance (sensitivity, specificity, predictive values and diagnostic likelihood ratios) was calculated only for participants where a definitive HIV diagnosis (reference-standard) was available, using the “diagt” Stata component. Because predictive values are affected by the prevalence of disease in the tested population, we also report the positive and negative diagnostic likelihood ratios (DLRs), which are less affected by prevalence [17]. DLRs incorporate both the sensitivity and specificity of a test and provide a direct estimate of how much a test result will change the odds of having or not having a disease. DLR calculation uses the following formulae:
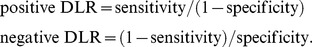



Positive DLRs can thus range from unity to infinity, negative DLRs from unity to 0 with better tests having DLRs further away from unity.

Uni-variable and multi-variable binomial regression with a log link adjusted for within household clustering was used to calculate risk ratios for possible associations of false positive RDT results with participant age, sex, *P. falciparum* and *S. haematobium* infection status, and elevation and annual mean ambient temperature at the participant’s residence.

## Results

During the first survey we obtained Determine RDT results from 8,747 female and 7,590 male participants between 2 and 97 years of age with a median age of 19.8 years (inter-quartile-range [IQR]  = 10.2 to 35.3) and 16.8 years (IQR  = 9.2 to 34.1) respectively. Of these RDT results 1,889 were obtained using whole blood samples (median age  = 4.1 years, range 2 to 87, IQR  = 2.9 to 5.3 years) and 14,448 using plasma (median age  = 21.1 years, range 2 to 97, IQR  = 12.1 to 36.9). Samples that could not be verified according to the above reference algorithm were excluded. Therefore, the below analysis was done with 1,696 and 12,916 Determine RDT results, obtained from testing whole blood and plasma samples respectively (Figure 1).

During the second survey the STAT-PAK RDT was used instead of the Determine RDT and we collected 2.7 ml of blood by venae puncture from all participants above the age of 2 years, so that testing with whole blood was no longer necessary. We obtained STAT-PAK RDT results from 8,574 female and 7,383 male participants with a median age of 19.1 (range  = 2 to 98, IQR  = 9.9 to 35.6) and 16.1 (range  = 2 to 96, IQR  = 8.8 to 34.0) years respectively. Again we excluded all samples where verification of the result was not possible which resulted in performing the analysis with 13,139 STAT-PAK RDT results, all derived from plasma samples (Figure 2).

### Determine RDT

The sensitivity and specificity of the Determine RDT were 100% and 96.8% in whole blood and 100% and 97.9% in plasma respectively. We did not encounter any false negative results with this test (tables 1 and 2). However, the positive predictive value was low in plasma (82.6%) and very low in whole blood (32.9%), meaning that 67.1% of the positive results were incorrect in whole blood and 17.4% in plasma. This high proportion of false positive tests was mainly due to the appearance of faint bands. Only 2% of whole blood samples with faint bands were truly HIV-1 positive according to the reference-standard. In plasma the proportion of true positive faint bands was 7.6%. For those whole blood and plasma samples where strong bands were recorded the concordance with the reference-standard was 86.2% and 96.4% respectively.

**Table 1:** Diagnostic Test performance of Determine and STAT-PAK RDT numbers of positive and negative results.

HIV RDT:	Determine		STAT-PAK
tested sample:	whole blood	plasma	plasma
N included	1696	12916	13139
Reference standard positives	26	1184	1170
Reference standard negatives	1670	11732	11969
True positives, all	26	1184	1166
True positives, faint bands	1	17	55
True positives, strong band	25	1167	1111
False positives, all	53	250	87
False positives, faint band	49	207	66
False positives, strong band	4	43	21
True negatives	1617	11482	11882
False negatives	0	0	4

**Table 2:** Diagnostic Test performance of Determine and STAT-PAK RDT sensitivity, specificity, predictive values and likelihood ratios.

	Determine RDT in whole blood		Determine RDT in blood plasma		STAT-PAK RDT in blood plasma	
	Result	95%CI	Result	95%CI	Result	95%CI
HIV prevalence according to reference standard^a^ (%)	1.53	1.00 to 2.24	9.17	8.67 to 9.68	8.90	8.42 to 9.40
Sensitivity (%)	100.00	86.77 to 100.00	100.00	99.69 to 100.00	99.66	99.13 to 99.91
Specificity (%)	96.83	95.87 to 97.61	97.87	97.59 to 98.12	99.27	99.10 to 99.42
Positive predictive value (%)	32.91	22.75 to 44.40	82.57	80.50 to 84.50	93.06	91.51 to 94.40
Negative predictive value (%)	100.00	99.77 to 100.00	100.00	99.97 to 100.00	99.97	99.91 to 99.99
ROC area	0.984	0.980 to 0.988	0.989	0.988 to 0.991	0.995	0.993 to 0.996
Positive likelihood ratio	31.51	24.18 to 41.07	46.93	41.51 to 53.05	137.10	111.20 to 169.04
Negative likelihood ratio^b^	0.00	– to –	0.00	– to –	<0.01	0.00 to 0.01

a Prevalences differ from total population prevalence due to exclusion of participants.

b No confidence interval can be calculated if sensitivity  = 100%.

### STAT-PAK RDT

The sensitivity and specificity of the STAT-PAK RDT were 99.7% and 99.3% respectively; the positive and negative predictive values were 93.1% and 99.97%. Of the 11,886 samples with a negative STAT-PAK RDT result, four were positive according to ELISA and Western Blot testing. All four tests had a positive result in the previous and the following survey but were repeatedly negative when retested using the STAT-PAK RDT. Of the 1,253 positive STAT-PAK RDT results 9.7% showed a faint band, and again the proportion of true positives was lower in faint-banded (45.5%) than in strong-banded tests (98.1%). Of the positive RDT results, 1166 (93.1%) were confirmed with the reference algorithm.

### Comparison of Determine and STAT-PAK Performance

The 250 false positive plasma samples from survey 1 were retested with the Determine RDT in order to exclude technical errors. The Determine RDT was repeatedly false positive in all samples. To compare the performance of the two RDTs in false positive samples, we later re-tested 25 (10%) of these false positive plasma samples from survey 1 with the STAT-PAK RDT. None of these samples was positive using the STAT-PAK RDT. The 250 participants with false positive plasma samples in Survey 1 were followed closely in the consecutive surveys. Thirty-six did not join the surveillance in survey 2, of the remaining 214 participants only 2 had a false positive test result again using the STAT-PAK RDT. However, in other survey 2 participants the STAT PAK RDT was actually false positive, demonstrating a different pattern of false positivity for both tests. In order to investigate whether false-positivity might be a transient problem or a feature associated with the Determine RDT, samples of 19 participants who had a false positive result in survey 1 were re-tested in survey 2 both with the STAT PAK and the Determine RDT. It appeared that the false positive result of the Determine RDT was not transient, but persisted in 10 out of 19 cases (52.6%) whereas STAT-PAK RDTs performed on the same samples were negative in all these cases.

Comparing the Determine RDT results which were obtained in plasma, with those of the STAT-PAK RDT, we found a higher positive predictive value and a higher specificity of the STAT-PAK RDT. Four false negative results however lead to a lower sensitivity of the latter test. Comparing the overall performance of both tests we found a positive DLR of 46.9 for the Determine RDT and of 137.1 for the STAT-PAK RDT, when using plasma for both tests. This means that in our study population the initial likelihood to be HIV-infected increases 46.9 times with a positive Determine RDT and 137.1 times after receiving a positive result with the STAT-PAK RDT when these tests are done with plasma samples.

### Possible Influence of Concomitant Infections

We hypothesized that false positive results for both RDTs might be related to exposure to infectious agents other than HIV. Therefore we examined the associations of co-infection with *S. haematobium (urinary* schistosomiasis) and *P. falciparum (*malaria tropica), participant age, sex, altitude and ambient temperature at the site of the participant’s home with false positivity (coded as 0 =  RDT true negative; 1 =  RDT false positive) as the binary outcome, using only results obtained in plasma samples. The binomial regression models (tables 3 and 4) showed significant associations of false-positivity with age, both for the Determine RDT (probability ratio (PR) per 10 years increase in age  = 1.09; 95% confidence interval (95%CI)  = 1.03 to 1.15) and for the STAT-PAK RDT (PR  = 1.10; 95%CI  = 1.00 to 1.21), indicating that the probability of a false positive result in both tests increases by about 10% with a 10 years increase in age. Furthermore, false positive results of the Determine RDT were associated with low altitude (PR per 100 m increase  = 0.78; 95%CI  = 0.66 to 0.93). Associations of Determine RDT false positivity with ambient temperature and *P. falciparum* co-infection, which were significant in uni-variable regression, both became non-significant in the multi-variable analysis when altitude of residence was included into the model.

**Table 3:** Association of various factors with false positive Determine RDT results in plasma; uni- and multi-variable log-link binomial regression results adjusted for clustering within household (N = 11732).

	uni-variable			multi-variable*		
Covariatestratum	PR	95%CI	P	PR	95%CI	P
**Age**						
per 10 years	1.10	(1.04 to 1.16)	0.001	1.09	(1.03 to 1.15)	0.002
**Elevation**						
per 100 meters	0.88	(0.86 to 0.90)	<0.001	0.78	(0.66 to 0.93)	0.006
**Ambient temperature**						
per ^o^C	1.26	(1.20 to 1.32)	<0.001	0.81	(0.59 to 1.10)	0.180
***P. falciparum*** ** infection**						
negative	1	-	-	1	-	-
positive	1.93	(1.07 to 3.47)	0.028	1.47	(0.81 to 2.68)	0.210
**Gender**						
female	1	-	-			
male	1.00	(0.79 to 1.27)	0.992			
***S. haematobium*** ** infection**						
negative	1	-	-			
positive	0.99	(0.58 to 1.69)	0.980			

PR  =  probability ratio for a false positive result; p  =  p-value; 95% CI  = 95% confidence interval.

* covariates were only included in multi-variable model if uni-variable p-value <0.2.

**Table 4:** Association of various factors with false positive STAT-PAK RDT results in plasma; uni- and multi-variable log-link binomial regression results adjusted for clustering within household (N = 11969).

	uni-variable			multi-variable*		
Covariatestratum	PR	95%CI	P	PR	95%CI	p
**Age**						
per 10 years	1.11	(1.01 to 1.21)	0.035	1.10	(1.00 to 1.21)	0.045
**Gender**						
female	1	-	-	1	-	-
male	0.63	(0.41 to 0.96)	0.031	0.64	(0.42 to 0.98)	0.038
**Elevation**						
per 100 meters	1.02	(0.97 to 1.06)	0.444			
**Ambient temperature**						
per ^o^C	0.99	(0.92 to 1.07)	0.808			
***S. haematobium*** ** infection**						
negative	1	-	-			
positive	0.62	(0.15 to 2.52)	0.502			

PR  =  probability ratio for a false positive result; p  =  p-value; 95% CI  = 95% confidence interval.

*P. falciparum* infection not included due to empty cells (no false positive results in *P. falciparum* infected participants because of low *P. falciparum* prevalence in survey 2).

* variables were only retained in multi-variable model if uni-variable p-value <0.2.

As opposed to the Determine RDT, false positive STAT PAK results were also associated with participant sex, with males having a significantly lower proportion of false positive results than females (PR  = 0.63, 95%CI  = 0.42 to 0.98).

## Discussion

An essential requirement of all HIV testing is the accuracy of the test results. HIV RDTs are usually designed to be used as screening tests with a positive result being confirmed by more specific methods. Therefore they are geared towards high sensitivity. This is reflected in the results of both RDTs that we evaluated: when read according to manufacturer’s instructions, the Determine RDT was 100% sensitive and the sensitivity of the STAT-PAK RDT was very close to this optimum. However, both tests had problems regarding specificity, which – for the Determine RDT – was worse when using whole blood instead of plasma for testing.

According to a WHO publication from 2002, the sensitivity and specificity of the Determine RDT in whole blood was 100% and 99.7% respectively [18]. Studies from the Ivory Coast [10], the Central African Republic [11], Cameroon [19], Tanzania [20], [21] and the Netherlands [12] reported slightly lower sensitivities, but similar specificity when using plasma [10], [11], [12] or whole blood [19], [20], [21]. However, two recent publications from Uganda demonstrated lower than expected specificities of 94% [7] and 91.7% [8] for the Determine RDT.

One possible reason for these diverse reports could be the subjective interpretation of test results [22]. A recent study from Uganda suggests that the difference in reported specificity of the Determine RDT could be caused by different interpretation of faint positive bands [7]. The authors found a specificity of 94.1% for the Determine RDT in plasma, which improved to 99.6% after exclusion of faint positive bands. Our data confirm these findings in a much larger population and show that the problem is aggravated when using whole blood instead of plasma. The interpretation of faint positive bands as negative might thus be one reason for the differences in sensitivity and specificity of the Determine RDT that are reported in some of the previous studies.

Most studies that examine the diagnostic performance of HIV RDTs use plasma or serum for testing. However, another study from Uganda that used whole blood found a specificity of the Determine RDT of only 96.2% which is very similar to our results [23].

Because the STAT-PAK RDT results were exclusively derived from plasma samples we can only compare them to the plasma results of the Determine RDT. The STAT-PAK RDT was considerably more specific than the Determine RDT. However, still 6.9% of all positive test results were false and again the proportion of true positives was lower in faint-banded (45.5%) than in strong-banded tests (98.1%).

It is noteworthy that half of our participants with false positive Determine RDT results were still false positive when re-tested with the Determine RDT one year later. This shows that for these participants the cause of the false positive result remains present over at least 12 month. It has been suggested that the laboratory diagnosis of HIV might be confounded by regional factors, for example cross reaction with antibodies against other diseases such as schistosomiasis [24]. We demonstrated that in our study area with an altitude range from 475 to over 2300 meters, false positive results of the Determine RDT, but not the STAT-PAK RDT, are more common in the lower parts with higher ambient temperatures that favour co-infections with various infective agents [25], [26]. In contrast to the published data regarding the Murex HIV Ag/Ab Combination EIA [24] we did not find an association with concurrent schistosomiasis infection for any of the rapid tests. Results in the uni-variable analyses show an association of Determine RDT false positivity with *P. falciparum* infection with nearly twice as many false positive Determine results in participants who were co-infected with *P. falciparum*. However, in multi-variable analysis, which included altitude of residence as an independent variable, this association became non-significant, suggesting that the cause of the false positive result was not *P. falciparum* infection. Similarly ambient temperature became non-significant when adjusted for altitude. It might be that in low altitude regions of our study area, where several other infections are common [27], humoral immune responses against concomitant infections interfere with the Determine RDT result. These potential cross-reactions could explain some of the differences between previous reports regarding the diagnostic performance of the Determine and other RDTs.

Interestingly we found no such association for temperature, altitude or co-infection with *P. falciparum* for the Stat-Pak RDT false positivity. The gender difference showing less false positive STAT PAK results in men than in women was not reported previously. This might be due to the fact that most studies focus on either women (microbicide trials) or men (circumcision trials). But we presently do not have an explanation for this finding.

Since age is a proxy for lifetime exposure to different diseases, cross reactions with persisting antibodies to other diseases should occur more frequently in older people, which is compatible with the rise of false positivity with age which is apparent in our data.

The relatively high number of false positive results that we found for both tests may not be a problem in settings where every positive screening result is confirmed by more specific, reliable and expensive methods. It is however problematic in resource constrained VCT settings, where testing is done on whole blood and where positive RDT screening results are usually confirmed only by additional RDTs, which might be susceptible to the same characteristics that caused the initial false positive result.

Since most of our false positive results showed faint bands, it is an important question how to deal with these. Interpreting them as negative is problematic as it would decrease sensitivity (e.g. from 100% to 98.6% for Determine RDT testing of plasma in our study) and classify some HIV-infected patients as uninfected with all the negative consequences this would have for transmission and the management of their infection. Interpreting them as positive would also be problematic as it would further decrease specificity of the test and cause unnecessary anxiety in some patients. We therefore believe that the best option in a screening setting is to record the strength of the band when testing and to consider this information once the standard testing reference algorithm for a positive sample is finalised. Positive results with faint bands should therefore be considered for additional testing in one of the Care and Treatment Centres, which have access to more specific methods, such as ELISA, Western Blot or PCR testing.

To avoid too many questionable results it would be best to chose a testing strategy which leaves as few open questions as possible. Thus it seems more sensible to use the Determine RDT with its high sensitivity but low specificity during initial screening and not as a confirmatory test as it was practiced until recently. For confirmation, a more specific test such as the Stat-Pak RDT should be used.

A strength of this study is its huge sample size and the fact that it includes male and female participants of all age groups, apart from infants. To our knowledge it is the first study to date to confirm the findings from Uganda showing that faint bands pose a problem when using the Determine RDT with plasma [7]. It is the only study which shows that this problem also occurs when using the STAT-PAK RDT and is aggravated when using whole blood. The two main limitations of this study concern the lack of direct confirmation (i.e. retesting of the same sample by other means) of negative results in whole blood and in plasma and of positive results obtained with whole blood. As mentioned above, RDT-negative samples were only retested if the RDT-result of the previous or the next survey was in disagreement ( =  positive) and regarded as reference-standard-negative if the result in the following survey was also negative. This could have led us to overestimate the sensitivity of both tests because we can not exclude that both the Determine and the STAT-PAK RDT are repeatedly false-negative in the same person. However, we believe that this would occur only very rarely, if at all. This is supported by the fact that none of the Determine-negative plasma samples that needed to be retested due to a positive RDT result in the next survey were regarded as positive according to our testing reference algorithm.

Because retesting of positive Determine RDT results obtained from whole blood samples was impossible due to lack of plasma, these were regarded as true positive if the result of the following survey was confirmed positive, as false positive if the result of the following survey was confirmed negative and excluded from this analysis if no HIV results from later surveys were available. This could have increased our specificity estimates for the Determine RDT in whole blood if initially HIV negative participants with a false positive Determine RDT result got HIV infected before the following survey. Equally it could have decreased our specificity estimates if an initial positive Determine RDT result was due to technical error. Although both is possible, it is again unlikely that this has happened in more than a few cases which would only marginally change the numbers presented here. Furthermore the specificity of the Determine RDT in whole blood is so low that a slight change in this diagnostic parameter would not make any difference in practice.

To summarize the above: Due to small shortcomings in our reference standard we can not completely exclude that the true sensitivities for both tests are slightly lower than reported in this article and that the true specificity of the Determine in whole blood may slightly deviate from the result reported here. However, both is unlikely, and with the large number of tests that were done, the very few cases where the reference standard might be incorrect would not substantially change the presented results.

### Conclusion

The Determine RDT strip test can not be recommended as a confirmatory test for prevalence studies and in VCT settings, due to its low specificity and low positive predictive value. The specificity of the test is even lower when using whole blood instead of plasma. However, with its optimal sensitivity of 100%, the Determine RDT is an excellent screening test, if another more specific RDT is used for confirmation of positive test results. Since our study did not apply the HIV-testing algorithm as stipulated in the National guidelines for voluntary counselling and testing in Tanzania, we are unable to judge the diagnostic performance of this algorithm. Nevertheless, the practice of using the Determine RDT as confirmatory assay in voluntary counselling and testing settings, where whole blood is used, should be reconsidered. The HIV 1/2 STAT-PAK RDT performed much better regarding specificity and was only slightly less sensitive. However, the problem of faint positive bands with their low positive predictive value could also be demonstrated for the STAT-PAK RDT, although to a far lesser extent.
